# Effect of Angiotensin System Inhibitors on Physical Performance in Older People – A Systematic Review and Meta-Analysis

**DOI:** 10.1016/j.jamda.2020.07.012

**Published:** 2021-06

**Authors:** Lorna Caulfield, Philip Heslop, Katherine E. Walesby, Deepa Sumukadas, Avan A. Sayer, Miles D. Witham

**Affiliations:** a AGE Research Group, NIHR Newcastle Biomedical Research Center, Newcastle University and Newcastle upon Tyne Hospitals NHS Foundation Trust, Newcastle upon Tyne, UK; b Alzheimer Scotland Dementia Research Center and Center for Cognitive Aging and Cognitive Epidemiology, University of Edinburgh, Edinburgh, UK; c Department of Medicine for the Elderly, NHS Tayside, Dundee, UK

**Keywords:** Systematic review, physical performance, sarcopenia, angiotensin converting enzyme inhibitor, angiotensin receptor blocker

## Abstract

**Objective:**

Preclinical and observational data suggest that angiotensin converting enzyme inhibitors (ACEi) and angiotensin receptor blockers (ARBs) may be able to improve physical performance in older people via direct and indirect effects on skeletal muscle. We aimed to summarize current evidence from randomised controlled trials in this area.

**Design:**

Systematic review and meta-analysis.

**Setting and Participants:**

Randomized controlled trials enrolling older people, comparing ACEi or ARB to placebo, usual care or another antihypertensive agent, with outcome data on measures of physical performance.

**Methods:**

We searched multiple electronic databases without language restriction between inception and the end of February 2020. Trials were excluded if the mean age of participants was <65 years or treatment was targeting specific diseases known to affect muscle function (for example heart failure). Data were sought on measures of endurance and strength. Standardized mean difference (SMD) treatment effects were calculated using random-effects models with RevMan software.

**Results:**

Eight trials (952 participants) were included. Six trials tested ACEi, 2 trials tested ARBs. The mean age of participants ranged from 66 to 79 years, and the duration of treatment ranged from 2 months to 1 year. Trials recruited healthy older people and people with functional impairment; no trials specifically targeted older people with sarcopenia. Risk of bias for all trials was low to moderate. No significant effect was seen on endurance outcomes [6 trials, SMD 0.04 (95% CI –0.22 to 0.29); *P* = .77; I^2^ = 53%], strength outcomes [6 trials, SMD –0.02 (95% CI –0.18 to 0.14), *P* = .83, I^2^ = 21%] or the short physical performance battery [3 trials, SMD –0.04 (95% CI –0.19 to 0.11), *P* = .60, I^2^ = 0%]. No evidence of publication bias was evident on inspection of funnel plots.

**Conclusions and Implications:**

Existing evidence does not support the use of ACE inhibitors or angiotensin receptor blockers as a single intervention to improve physical performance in older people.

Impaired physical performance, exemplified by reduction in strength and endurance, is common with increasing age and with the multimorbidity that often accompanies age.^1^ Impaired physical performance leads in turn to a loss of the ability to perform activities of daily living, a need for care, and is associated with future disability, hospital admission, longer length of stay, and earlier death.^2^, ^3^, ^4^ Although exercise training is well established as a key therapy to improve physical performance in older people, not all older people are either willing or able to undertake exercise therapy. Alternative ways to improve physical performance in older people are therefore needed.

Angiotensin converting enzyme inhibitors (ACEi) and angiotensin receptor blockers (ARB) are classes of medication that work by either inhibiting production of angiotensin II or blocking the effect of angiotensin II at the AT1 receptor. They have a number of beneficial effects on cardiovascular physiology including improved endothelial function, reduced myocardial fibrosis, regression of left ventricular hypertrophy, and improvement of left ventricular systolic function. Use of these medication classes improves function and prognosis in a wide range of cardiovascular conditions including heart failure, hypertension, stroke, and ischemic heart disease.^5^, ^6^, ^7^, ^8^

More recently, a number of biological mechanisms have been elucidated by which these drugs might improve peripheral skeletal muscle function.^9^ Angiotensin II has direct deleterious effects on skeletal muscle structure and function in experimental conditions^10^^,^^11^ and may impair both macrovascular and microvascular endothelial function, and hence, blood flow in peripheral vascular beds.^12^ Angiotensin II also promotes chronic inflammation,^13^ which is in turn thought to be an important driver of sarcopenia—the age-related loss of muscle mass and strength that underpins impaired physical performance in many older people. Conversely use of ACEi and ARBs can ameliorate these deleterious effects in experimental conditions; ACEi or ARB treatment reduces inflammation and endothelial dysfunction in hypertension^14^^,^^15^ and can improve skeletal muscle atrophy.^16^ In addition, ARBs have been shown to augment the effect of exercise on suppression of myostatin, a key inhibitor of the hypertrophic response to exercise.^17^ Finally, ACEi can improve glucose uptake by skeletal muscle by augmenting insulin function in peripheral tissues.^18^

A number of randomized control trials have been conducted to examine the effects of ACEi and ARBs on skeletal muscle function in older people. Results have been mixed but only 1 previous systematic review has attempted to synthesize these data.^19^ This systematic review was conducted in 2015 and included only 4 studies. Since then, a number of other studies have been published. The aim of this analysis was, therefore, to conduct an up-to-date and thorough systematic review of the effect of ACE inhibitors and angiotensin receptor blockers on both endurance and strength performance in older people.

## Methods

The review protocol was prespecified and registered on the PROSPERO database (registration number CRD42014013398). The review was reported using PRISMA statement guidance.^20^

### Search Strategy and Selection Criteria

We searched electronic databases (Medline, CINAHL, Embase, Cochrane Central Register of Controlled Trials, Controlled Clinical Trials.com, and NHS elibrary) between inception and the end of February 2020. No date or language restrictions were employed. An example search string is shown in Appendix 1. Reference lists of included studies were hand-searched for additional candidate trials.

We included randomized controlled trials involving human participants with a mean age of 65 years or over. Trials had to study ACE inhibitors or ARBs, given for a minimum of 4 weeks. Comparators could include usual care, placebo, or another class of antihypertensive. Co-interventions were permitted if the co-intervention (eg, exercise training) was applied to both the ACEi/ARB arm and the comparator arm.

We excluded trials performed for specific disease states known to impair exercise capacity via mechanisms other than by effects on skeletal muscle (for instance ischemic heart disease, heart failure, chronic obstructive pulmonary disease), which may limit exercise capacity via cardiorespiratory compromise, and also via a specific type I muscle fiber skeletal myopathy.^21^^,^^22^ Trials focusing on hypertension were permitted, as were trials focusing on people with diabetes or obesity. We further excluded trials where an ACEi was compared with an ARB.

### Data Collection and Extraction

Two reviewers (L.C. and M.W.) reviewed all titles after deduplication of the search results. Titles flagged as requiring further scrutiny by either reviewer had abstracts retrieved. Both reviewers reviewed the retrieved abstracts, and full text papers were flagged by either reviewer were retrieved. Papers agreed as eligible by all 3 reviewers (L.C., P.H., M.W.) were forwarded for data extraction. Data were extracted using a standard, piloted form. One reviewer (L.C. or P.H.) extracted data, which was then checked by M.W.

We extracted baseline data on trial populations (including age, sex, functional status, comorbidities, and blood pressure), intervention type, dose and duration, and details of cointerventions. We sought a wide range of measures of physical performance, broadly classified as measures of endurance (including, but not limited to 6-minute walk distance, 12-minute walk, cycling time, VO_2_ max, incremental shuttle walk test, seated step test, arm curl test, recovery heart rate, or treadmill endurance time), or measures of strength/power [including, but not limited to sit-to-stand test, handgrip strength, leg (quadriceps) strength, timed up and go test (TUG), stride length, short course gait speed, jump height]. We sought data on the Short Physical Performance Battery (SPPB) as a specific outcome. For all outcomes, the longest available follow-up treatment point was included in analyses if more than 1 time point during treatment was reported.

### Assessing Methodological Quality of Included Studies

Risk of bias for each trial was independently assessed by 2 reviewers (L.C. and M.W.) using the following categories: allocation concealment, description of withdrawals and dropouts, analysis on intention to treat, participant, healthcare staff and outcome assessor blinding, and comparability of treatment groups at baseline. Trials were judged as either as low risk, unclear, or high risk.^23^ Disagreements were resolved by discussion.

### Meta-Analysis

Data were combined in meta-analyses using RevMan 5.3 software (Cochrane Collaboration, Copenhagen, Denmark) using weighted-squares methods. Random effects models were used for all analyses to ensure a conservative approach to calculating 95% confidence intervals given the likely heterogeneity of interventions and populations.

For endurance measures, the 6-minute walk distance was used as the first choice, followed by other walk distance tests, then exercise time, then other tests (eg, VO_2_max) if no other data were available. Similarly, for strength tests, quadriceps strength was used as the first choice, followed by handgrip strength, then timed up and go or sit to stand tests. SPPB results were combined in a separate meta-analysis as these are composite tests of balance, walk speed, and leg strength.

Analyses were reported using standardized mean differences (SMDs) where more than 1 outcome measure type was combined. Change scores and standard deviation (SD) of change were used where reported; percentage change (and SD of percentage change) was used if this was available in the absence of raw change scores. Where only baseline and follow-up data were available, change scores were calculated as the difference between mean follow-up and mean baseline scores, and the mean of baseline and follow-up standard deviation was used as a measure of variance. For crossover trials, adjustment of the standard error was performed as previously recommended^24^ to ensure adequate weighting of the study in the analysis. Funnel plots were generated and inspected visually for asymmetry suggesting possible publication bias. Sensitivity analyses confined to homogenous outcomes were performed, along with analyses using the first available follow-up time point as opposed to the last available follow-up time point to test for early treatment effects and to mitigate the effect of dropout with time.

## Results

**Fig. 1:**
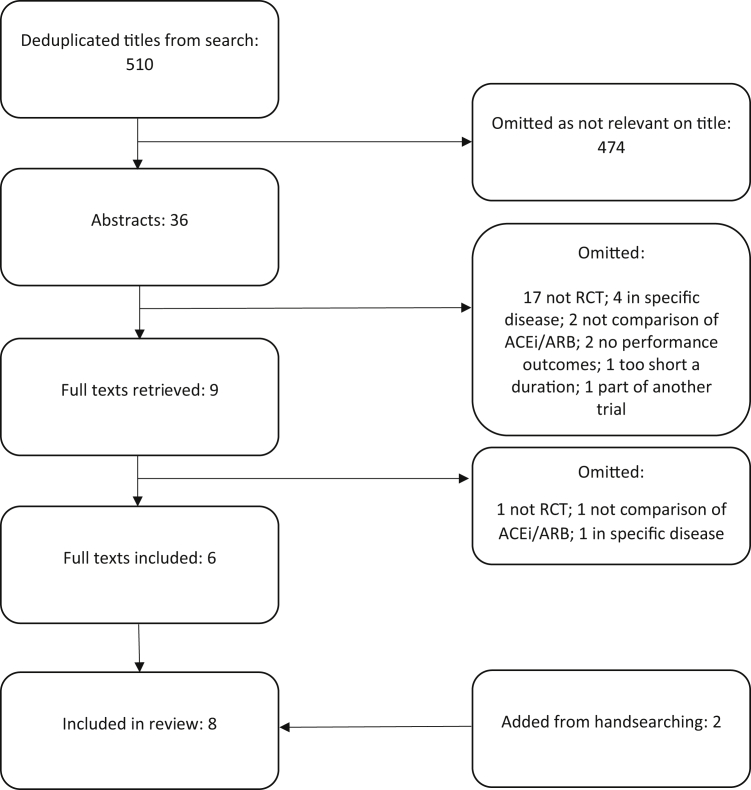
PRISMA flow diagram. RCT, randomized controlled trial; SR, systematic review.

The de-duplicated search found 510 titles; 6 of these were included in the systematic review, along with 2 other studies found during hand searching of references. The PRISMA flow diagram is shown in Figure 1. The 8 studies included a total 952 participants, with mean ages ranging from 66 to 79 years.

Table 1 shows details of the included studies.^25^, ^26^, ^27^, ^28^, ^29^, ^30^, ^31^, ^32^ Three trials included participants with functional impairment, 4 trials included older people with hypertension or elevated cardiovascular risk, and 1 trial included healthy older men. No trials specifically aimed to recruit participants with sarcopenia or frailty. Trial size ranged from 36 to 294, with four trials enrolling more than 100 participants. The agents studied varied; ACEi in 6 studies and an ARB in only 2 studies. In 2 trials, an alternative antihypertensive was used as a comparator; placebo was used in the other trials. The duration of treatment varied from 15 weeks to 1 year. Two trials examined the effect of ACEi or ARBs in augmenting the effect of background exercise training. Supplementary Table 1 shows all outcomes reported for each included trial.

**Table 1:** Details of Included Studies

	Country	n	Mean Age, y	% Women	Inclusion Criteria	Baseline Function	Intervention	Comparator	Primary Outcome	Secondary outcomes	Duration of Treatment
Leonetti 1991^25^	Italy	36	66	72	Older people with hypertension	Cycle endurance time 536 s	Captopril 25‒50 mg twice daily	Placebo	Bicycle endurance exercise time	None	2 mo
Gerdts 2006^26^	Norway	51	68	49	55‒80 y with hypertension and LVH on ECG	VO_2_max 23.7 mL/kg/min Maximal load 120W	Losartan 50-100 mg once daily + HCTZ if required	Atenolol 50‒100 mg once daily + HCTZ if required	VO_2_max	Maximum load (W)	1 y
Sumukadas 2007^27^	Scotland	130	79	71	65 and over with impairment of ADLs	Mean 6MWD 299 m Median TUAG 13s Median 10-rep STS 37 s	Perindopril 2-4 mg once daily	Placebo	6MWD	TUAG 10-rep STS	20 wk
Bunout 2009^28^	Chile	120	75	76	70 and over with stage I hypertension	Mean 12MWD 916 m Mean grip strength 23.5 kg Mean quads strength 27.3 kg Mean SPPB 9.2 Mean TUAG 11.3 s	Enalapril 10‒20 mg once daily + HCTZ if required	Nifedipine slow-release 20 mg once daily	12MWD	Handgrip strength Quads strength SPPB TUAG	9 mo
Cesari 2010^29^	USA	294	66	42	55 and over with elevated cardiovascular risk	Rescaled SPPB Handgrip 39.0 kg	Fosinopril 20‒40 mg once daily	Placebo	Rescaled SPPB	Handgrip strength	6 mo
Sumukadas 2013^30^	Scotland	170	76	42	65 and over with SPPB ≤10	Mean 6MWD 306m Mean grip strength 20.1 kg Mean quads strength 18.4 kg Mean SPPB 7.6	Perindopril 2‒4 mg once daily + mixed modality exercise training	Placebo + mixed modality exercise training	6MWD	SPPB Quads strength Handgrip strength	20 wk
Sumukadas 2018^31^	Scotland	80	78	75	65 and over with >1 self-reported fall in last 12 mo	Mean 6MWD 333 m Mean quads strength 18.9 kg	Perindopril 2‒4 mg once daily	Placebo	Postural sway	6MWD Quadriceps strength	15 wk
Heisterberg 2018^32^	Denmark	71	72	0	Healthy, untrained male persons without hypertension or other disease	Mean 1-rep max quads strength 83 kg	Losartan 50‒100 mg once daily + resistance training	Placebo + resistance training	Quadriceps mass	Isometric Quadriceps strength Isokinetic quadriceps strength	16 wk

ADL, activities of daily living; 6MWD, 6-minute walk distance; 12MWD, 12-minute walk distance; STS, sit to stand test; TUAG, timed up and go test; VO_2_max, maximal oxygen uptake.

### Quality Assessment

**Supplementary Fig. 1:**
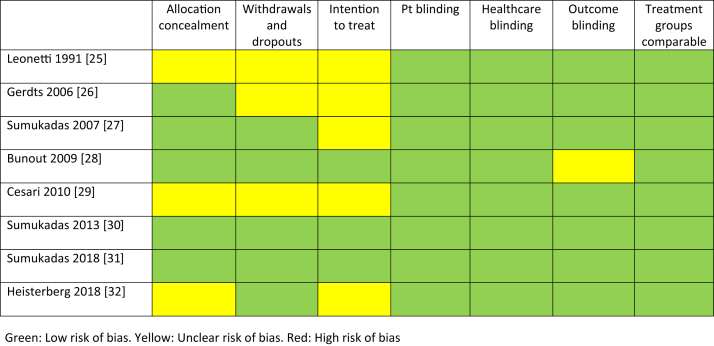
Risk of bias assessment. Low risk of bias (*green*). Unclear risk of bias (*yellow*). High risk of bias (*red*).

**Supplementary Fig. 2:**
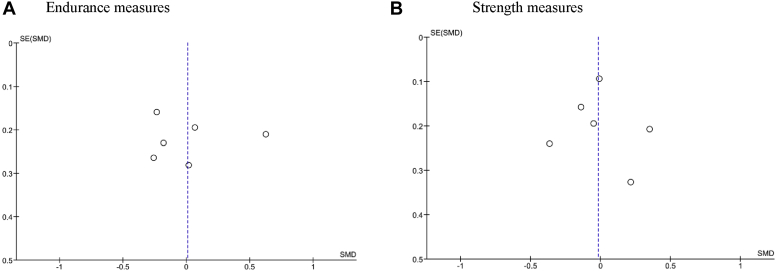
Funnel plots for risk of publication bias. (A) Endurance measures. (B) Strength measures.

Supplementary Figure 1 shows the risk of bias assessment for the included trials. The overall risk of bias was low; trials were blinded and generally well balanced for baseline characteristics. Allocation concealment and randomization methods were unclear or insufficiently detailed in some trials. Funnel plots for endurance and strength outcomes are shown in Supplementary Figure 2; these did not suggest publication bias.

### Effect on Endurance

**Fig. 2:**
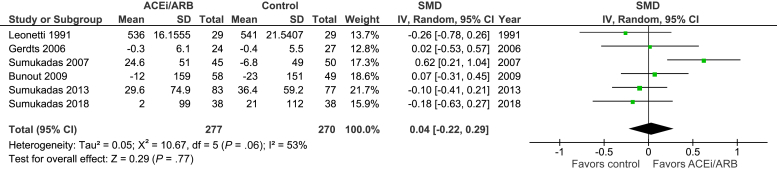
Endurance measures.

Figure 2 shows the pooled effect on endurance. Combining data from 6 trials (6-minute walk distance in 3, cycle endurance time, 12-minute walk distance and VO_2_max in another 3) showed no significant effect of ACEi on exercise capacity [standardized mean difference 0.04 (95% CI –0.22 to 0.29); *P* = .77; I^2^ = 53%, n = 547]. Confining the analysis to the 3 trials using 6-minute walk distance also showed no evidence of benefit [mean difference 5 m (95% CI –26 to 37); *P* = .74; I^2^ = 76%, n = 311]. In both cases, a small beneficial effect size (SMD >0.2 or 6 minute walk distance >20 m^33^) still lies within the 95% CIs. A further sensitivity analysis using measurements from baseline and the first available follow-up time also showed no evidence of benefit [SMD 0.12([95% CI –0.07 to 0.30); *P* = .23; I^2^ = 19%, n = 562].

### Effect on Strength

**Fig. 3:**
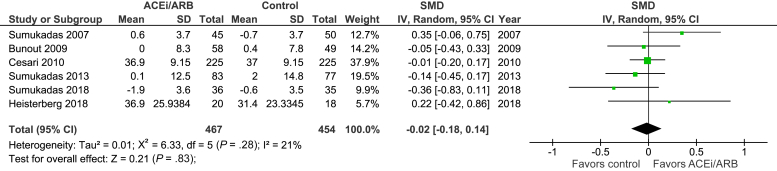
Strength measures.

Figure 3 shows the pooled effect on strength measures. Of the 6 included trials, 4 measured quadriceps strength (by a variety of different techniques), 1 measured handgrip strength, and 1 measured the timed up and go test. No significant beneficial treatment effect was evident [SMD –0.02 (95% CI –0.18 to 0.14), *P* = .83, I^2^ = 21%]. Excluding the cross-over trial^29^ did not change the results: [SMD –0.02 (−0.25 to 0.22); *P* = .89; I^2^ = 37%, n = 471]. When restricting the analysis to only those trials measuring quadriceps strength, no significant treatment benefit was seen [mean difference −1.1 kg([–2.5 to 0.2); *P* = .11. I^2^ = 0%, n = 376]. A further sensitivity analysis using measurements from baseline and the first available follow-up time also showed no evidence of benefit [SMD -0.01 (95% CI –0.16 to 0.14); *P* = .88; I^2^ = 17%].

### Effect on SPPB

**Fig. 4:**
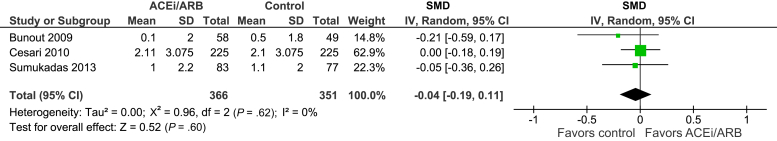
SPPB.

Figure 4 shows the pooled effect on the SPPB. Two included trials measured the SPPB using the standard 12-point scale; 1 trial used a rescaled approach to maximize the power of the continuous measurement components of the score.^29^ No significant beneficial treatment effect was evident [SMD –0.04 (95% CI –0.19 to 0.11), *P* = .60, I^2^ = 0%]. Excluding the cross-over trial^29^ showed similar findings [mean difference −0.2 points (−0.7 to 0.3); *P* = .34. I^2^ = 0, n = 267]. The minimum clinically important difference in the SPPB has been estimated at between 0.5 and 1 point^33^^,^^34^; both of these estimates lie outside the 95% CIs for the estimated treatment effect in this analysis. A further sensitivity analysis using measurements from baseline and the first available follow-up time also showed no evidence of benefit [SMD 0.03 (95% CI –0.12 to 0.18); *P* = .70; I^2^ = 0%]

## Discussion

### Summary of Evidence

Our systematic review found no evidence of efficacy of ACEi or ARB in improving either strength or endurance measures of physical performance in older people. Overall trial quality was moderate to good, but trials were in general small with few trials examining outcomes beyond 6 months. Heterogeneity was low; there appeared to be no benefit of ACEi or ARB either alone or in conjunction with exercise training. No difference was apparent in the efficacy of ACEi compared with ARBs, although head-to-head comparisons were not included in this analysis. Our findings are consistent with the previous meta-analysis conducted in 2015 by Zhou et el^19^ despite the inclusion of more trials, more participants, and more detailed analyses.

### Limitations

There are a number of limitations to our analysis. As with any systematic review, it is possible that we have omitted relevant literature although the use of a broad search strategy, no language restrictions, and inclusion of studies found by hand searching reduced the chances of missing significant literature. The scope of our review was limited to participants without a specific disease or condition affecting muscle strength. We made this choice in an attempt to focus on whether ACEi or ARBs might have an effect on impaired physical performance caused by sarcopenia of age and related problems, rather than by other skeletal myopathies related to specific disease states. Although the effect of ACEi or ARBs on physical performance in patients with heart failure, chronic obstructive pulmonary disease, or other cardiorespiratory disease is clearly of interest, study of these conditions with their distinct skeletal myopathy and prominent cardiorespiratory compromise falls out with the scope of the current analysis. Studies targeting patients with diabetes and obesity are of interest given the prominent association between these conditions and skeletal muscle dysfunction,^35^^,^^36^ but our search did not find eligible trials with relevant outcomes.

The small number of studies included, and the broad range of outcomes studied, made combining data in meta-analysis challenging. For most outcomes, we had to resort to reporting standardized mean differences because of this heterogeneity in outcomes. Perhaps the most important limitation of this review, however, is that none of the included studies specifically sought to recruit patients with sarcopenia as defined by contemporary guidelines. Although some of the studies undoubtedly included participants with sarcopenia (particularly those which sought to recruit patients with functional impairment), other studies aimed to recruit healthy older people. We cannot, therefore, presume that the lack of effect seen in this analysis necessarily applies to patients with a diagnosis of sarcopenia. The majority of studies included more women than men; this reflects both the predominance of women in the oldest old, and the fact that older women are more likely to have low physical performance. We are not able to examine any differential effects of ACEi or ARBs on men and women from this trial-level analysis. Additional limitations include a lack of data on ARBs that were studied in only 2 trials, and a lack of long-term outcome data; most studies were confined to less than 6 months follow-up. It is, therefore, possible that longer term use of ACEi or ARBs may still yield effects; earlier observational data from Onder et al^37^ suggested that differences in walking speed between users and nonusers of ACEi were evident after 3 years of follow-up, although more recent observational data did not find any association between either ACE inhibitor use and grip strength^38^ or a similar use and other measures of physical performance.

## Conclusions and Implications

### Implications for Practice

Existing evidence does not support the use of ACEi or ARBs as stand-alone therapies to improve physical performance in older people, either with or without a diagnosis of sarcopenia. Although these agents are generally safe and well tolerated in older people and are highly effective at improving cardiovascular outcomes, their use in older people should be restricted to reducing blood pressure, reducing the risk of cardiovascular events, or to improving symptoms and function in older people with heart failure.^39^

### Implications for Research

Further research in this area should focus on people with a diagnosis of sarcopenia made using contemporary criteria such as those recommended by the European Working Group on Sarcopenia.^40^ Although it is unlikely that use of ACEi or ARBs as single agents over the short term would prevent progression to sarcopenia, a preventive effect on declines in physical performance over the longer term cannot be ruled out and long-term follow-up from existing ACEi and ARB studies could still shed light on this. It is also still possible that combination treatment with these agents and others targeting complementary biological pathways in sarcopenia could yield benefits, although the evidence presented in this systematic review did not support a role in augmenting the effect of exercise. Future studies should endeavor to use a consistent and limited range of performance measures; hand grip strength, short physical performance battery, 6-minute walk, and quadriceps strength would give a set of core outcomes that would most easily combine with existing trial data, and would accord with recent recommendations for core outcomes in sarcopenia trials.^41^

## Figures and Tables

**Supplementary Table 1:** Outcome Measures Collected in Each Included Trial

	Endurance Measures					Strength Measures				
	6MWD	12MWD	VO_2_max	Maximal load	Cycle Exercise Time	Grip	Quadriceps Strength	SPPB	TUAG	STS
Leonetti 1991^25^					X					
Gerdts 2006^26^			X	X						
Sumukadas 2007^27^	X								X	X
Bunout 2009^28^		X				X	X	X	X	
Cesari 2010^29^						X		X∗		
Sumukadas 2013^30^	X					X	X	X		
Sumukadas 2018^31^	X						X			
Heisterberg 2018^32^							X			

6MWD, 6-minute walk distance; 12MWD, 12-minute walk distance; STS, sit to stand test; TUAG, timed up and go test; VO_2_max, maximal oxygen uptake.

∗ Modified version of SPPB.

## Supplementary Data

PRISMA Checklist
